# Characterization of α-L-Rhamnosidase and β-D-Glucosidase Subunits of Naringinase Immobilized on a Magnetic Polysaccharide Carrier

**DOI:** 10.3390/ijms26199813

**Published:** 2025-10-09

**Authors:** Joanna Bodakowska-Boczniewicz, Zbigniew Garncarek

**Affiliations:** Department of Biotechnology and Food Analysis, Faculty of Production Engineering, Wroclaw University of Economics and Business, Komandorska 118/120, 53-345 Wrocław, Poland; zbigniew.garncarek@ue.wroc.pl

**Keywords:** α-L-rhamnosidase, β-D-glucosidase, naringinase, immobilization, magnetic carrier

## Abstract

Naringinase consists of two enzymes: α-L-rhamnosidase and β-D-glucosidase. The enzyme was immobilized on a carrier prepared from carob gum activated with polyethyleneimine. Cross-linking with dextran aldehyde was used to improve the stability of the immobilization. Knowledge of the characteristics of naringinase subunits is important for developing efficient and selective enzymatic reactions involving flavonoids. This study aimed to characterize two subunits of naringinase—α-L-rhamnosidase and β-D-glucosidase—free, immobilized on a magnetic polysaccharide carrier and cross-linked with dextran aldehyde. The characterization of free, immobilized, and stabilized naringinase, as well as α-L-rhamnosidase and β-D-glucosidase, included the effect of pH and temperature on enzyme activity, as well as the determination of their stability depending on the pH and temperature of the environment, and the determination of kinetic constants. Immobilization and subsequent stabilization of naringinase did not affect the optimal pH for the activity of α-L-rhamnosidase and β-D-glucosidase. Immobilization caused a change in the optimal temperature for the activity of α-L-rhamnosidase and β-D-glucosidase from 60 to 65°. Cross-linking of immobilized naringinase with dextran aldehyde increased the temperature stability of its subunits. Cross-linking also altered the pH stability profile of β-D-glucosidase. Immobilization and stabilization of naringinase slightly reduced the maximum reaction rate for α-L-rhamnosidase and β-D-glucosidase compared to the free enzyme. As a result of immobilization, the enzymes’ affinity for substrates for both subunits decreased.

## 1. Introduction

Naringinase is an enzyme with dual activity: α-L-rhamnosidase (EC 3.2.1.40) and β-D-glucosidase (EC 3.2.1.21) [1,2]. Some authors define naringinase as an enzymatic complex with α-L-rhamnosidase and β-D-glucosidase activity [3,4]. In contrast, others consider it a mixture of two separate enzymes, α-L-rhamnosidase and β-D-glucosidase [5,6]. The exact arrangement and number of subunits may vary depending on the microbial source. Chen et al. [7] demonstrated that naringinase from *Aspergillus aculeatus* was a tetramer consisting of one α-L-rhamnosidase subunit and three β-D-glucosidase subunits Naringinase from *A. niger* is also a multimeric enzyme; it has two subunits [8]. Two β-D-glucosidases and one α-L-rhamnosidases were purified from the naringinase from *A. terreus* CECT 2663 [9]. So far, only the crystal structure of α-L-rhamnosidase has been elucidated. Site-directed mutagenesis studied key residues involved in enzymatic catalysis and rhamnose recognition. Acidic residues such as Asp567, Glu572, Asp579, and Glu841 were identified as crucial for hydrolysis, and their mutation was shown to reduce enzymatic activity significantly [3]. The detailed structure of naringinase remains unclear and requires further investigation. Puri [1] suggested that crystallization of naringinase from different sources will be necessary to determine their precise structures.

This enzyme plays a key role in citrus processing by hydrolyzing naringin to remove bitterness in citrus juice [1,2,10]. It is also applied to enhance the flavor of wines, fruit juices, and various beverages [1,2,10]. Additionally, it can increase flavonoid bioavailability and antioxidant capacity, serve in the production of sweeteners, and improve the quality of soy-based products [10]

Naringinase is widely used in the biotransformation of flavonoids, compounds of significant biological and therapeutic importance. Many flavonoids occur naturally in the form of glycosides, which significantly affects their biological properties, including bioavailability and solubility [5].The presence of saccharide groups can lead to changes in the bioavailability of the corresponding flavonoid aglycone, depending on the type of sugar, e.g., glucosides are absorbed faster than other types of glycosides, such as rhamnosides and rhamnoglucosides. Due to the difficult absorption of flavonoids, solutions aimed at increasing their bioavailability are known, e.g., through deglycosylation [5]. Thanks to the ability of naringinase to deglycosylate compounds containing α-rhamnose or β-glucose at the ends of the molecule, many natural glycosides such as naringin, rutin, hesperidin, and diosmin can be its substrates [11,12]. For example, two enzymes, α-L-rhamnosidase and β-D-glucosidase, are necessary for the deglycosylation of naringin, the dominant grapefruit juice flavonoid. The first, α-L-rhamnosidase, hydrolyses naringin to rhamnose and prunin. Then, β-D-glucosidase hydrolyses prunin to glucose and naringenin [13,14]. Hesperidin, present in orange juice, can be deglycosylated by α-rhamnosidase to rhamnose and 7-O-glucoside hesperetin (Hes-7-G), which is then hydrolyzed by β-D-glucosidase to glucose and hesperetin [15,16,17]. α-L-rhamnosidase from naringinase hydrolyses rutin (3-O-rutoside of quercetin) to rhamnose and 3-O-glucoside of quercetin (quercetin-3-O-glucoside Q-3-G or isoquercitrin) β-D-glucosidase, in turn, hydrolyses isoquercitrin to glucose and quercetin [11,18,19]. However, these flavonoids’ hydrolysis effectiveness depends on the enzymes’ stability and activity in the target environment, which is most often acidic fruit (pH 3–4).

Knowledge of the properties of individual naringinase subunits, their optimal operating conditions, and the influence of environmental conditions on their activity is crucial for targeted flavonoid hydrolysis. This knowledge enables control of the enzymatic reaction, e.g., through selective inactivation of one of the subunits to stop hydrolysis at the intermediate glycoside stage. Determination of the optimal pH and temperature conditions for the activity of enzyme subunits is essential for increasing the efficiency of deglycosylation processes involving naringinase. This approach allows for precise control of the flavonol hydrolysis process.

In the context of the practical application of naringinase, particularly in the food industry, its use in industrial conditions is also an important aspect. Using the free form of the enzyme poses several limitations, such as the inability to reuse it or the difficulty of separating the enzyme from the final product. In response to these challenges, enzyme immobilization techniques are becoming increasingly important, as they allow for reuse, improved stability, and simplification of the technological process [20,21]. Natural carriers such as proteins, porous glass, silica gel, and polysaccharides are used for enzyme immobilization. Polysaccharide-based materials, in particular, are a suitable matrix for enzyme immobilization due to many chemically active groups, a large specific surface area, and significant sorption capacity [22]. The use of such natural carriers for the immobilization of naringinase, such as carrageenan [23], chitosan [24], alginate [25,26,27] and corn cob powder [28], has been described in the literature. In order to increase the efficiency of enzyme binding to the matrix, carriers are often modified, e.g., by using aldehyde groups reacting with ion polymers containing amino groups, such as polyethyleneimine, or by activating the polymer skeleton containing amino groups with glutaraldehyde. Dextran aldehyde can be an additional cross-linking agent [21,29]. Recently, researchers have been particularly interested in the immobilization of enzymes on carriers with magnetic properties [30,31,32,33,34,35]. Thanks to a magnetic field, the most significant advantage of their use is the quick and easy separation of the enzyme from the reaction medium. To date, there have been few studies on the immobilization of naringinase on magnetic carriers [21,36,37,38]. The immobilization of naringinase often leads to increased stability under changing pH and temperature conditions and in solvent environments. Immobilization of naringinase can significantly affect its stability at different pH of the environment, often broadening and sometimes shifting the optimal range of enzyme activity [21,39,40]. According to some authors, immobilization of naringinase often contributes to an increase in the optimal temperature of activity of this enzyme [27,41,42]. The higher optimal temperature of naringin hydrolysis by naringinase increases the possibility of using the stabilized enzyme on an industrial scale [43]. There are a few reports in the literature on the effect of naringinase immobilization on the stability and optimal conditions for the activity of its subunits. Characterizing α-L-rhamnosidase and β-D-glucosidase, which are components of naringinase, is an important step towards designing effective and selective enzymatic processes involving flavonoids. Combining knowledge about the enzymatic characteristics of naringinase subunits with modern immobilization methods provides a solid basis for developing efficient, economical, and sustainable biotechnological processes.

Naringinase from *Aspergillus niger* KMS was adsorbed onto a carrier obtained from carob gum activated with polyethyleneimine. In order to strengthen the bond between the enzyme and the carrier, naringinase was additionally stabilized by cross-linking with dextran aldehyde [21]. This study aimed to investigate the effect of immobilization of naringinase on the optimal parameters of its subunits’ activity. Two subunits of naringinase were characterized, α-L-rhamnosidase and β-D-glucosidase, occurring in various forms: free, immobilized on a magnetic polysaccharide carrier, and additionally cross-linked with dextran aldehyde. The characterization includes determining the influence of pH and temperature on enzyme activity, their stability depending on the pH and temperature of the environment, and the determination of reaction kinetic constants. The influence of environmental conditions on the enzymatic activity of α-L-rhamnosidase and β-D-glucosidase is essential for their practical application. This knowledge will enable targeted deglycosylation of flavonoids, which will contribute to increasing their bioavailability.

## 2. Results and Discussion

### 2.1. Immobilization Yield

The immobilization efficiency of naringinase subunits on the magnetic polysaccharide carrier was calculated based on the total activity of this enzyme used for immobilization and the activity of the immobilized enzyme. The immobilization yield of α-L-rhamnosidase on the magnetic polysaccharide carrier activated with PEI was 18.09% relative to the total activity of this enzyme used for immobilization. In the case of β-D-glucosidase, the immobilization yield on the same carrier was 31.27%. The results indicate that α-L-rhamnosidase was immobilized with a lower yield than β-D-glucosidase.

This difference may result from conformational features of the two subunits, which affect their interaction with the functional groups of the PEI-activated polysaccharide carrier. Cross-linking of the immobilized enzymes likely caused further conformational changes, stabilizing the structure of the biocatalysts and contributing to increased activity. More significant changes occurred in the case of β-D-glucosidase.

Kimmis et al. [38], who immobilized naringinase from *A. aculeatus* and *A. niger* on polydopamine (PDA)-coated magnetic nanoparticles, obtained a similar immobilization efficiency for α-L-rhamnosidase of 19.3 ± 0.8%, while for β-D-glucosidase it was only 2.2 ± 0.1% of the total activity. Chemical amination of the enzyme significantly improved the immobilization efficiency, increasing the efficiency by more than 40% for α-L-rhamnosidase and more than 10-fold for β-D-glucosidase [38]. Wang et al. [44] using an artificial naringinase system (constructed by co-immobilization of α-L-rhamnosidase (*A. oryzae* FJ0123) and β-glucosidase (*Thermotoga maritima* MSB8) on magnetic silica-based chitosan microspheres) obtained a significantly higher immobilization efficiency, 61.4% for α-L-rhamnosidase and 90.1% for β-glucosidase, respectively.

### 2.2. Activity of α-L-rhamnosidase and β-D-glucosidase

Table 1 shows the enzymatic activity of two subunits of naringinase α-L-rhamnosidase and β-D-glucosidase in three different forms: free, immobilized by adsorption, and immobilized and additionally cross-linked.

The cross-linking with dextran aldehyde did not cause activity loss, as the measured values for both subunits remained nearly identical to those obtained after simple adsorption. Additional cross-linking primarily stabilizes the immobilized enzyme preparation without reducing its catalytic potential. The activity of α-L-rhamnosidase concerning β-D-glucosidase in the free naringinase complex was more than three times higher.

The proportional relationship between the activities of α-L-rhamnosidase and β-D-glucosidase was not preserved after immobilization, indicating that the functional balance of the naringinase complex was altered. The activity of α-L-rhamnosidase decreased relative to β-D-glucosidase. Immobilization may have induced greater conformational changes in the active site region of α-L-rhamnosidase, resulting in a reduced subunit activity compared to β-D-glucosidase. This observation is also supported by the differences in the immobilization yields of the two subunits.

The higher activity of α-L-rhamnosidase compared to β-D-glucosidase may be important in the selective production of compounds such as prunin, Hes-7-G or isoquercetrin [28].

### 2.3. Effect of pH on the Activity of Naringinase Subunits

**Figure 1:**
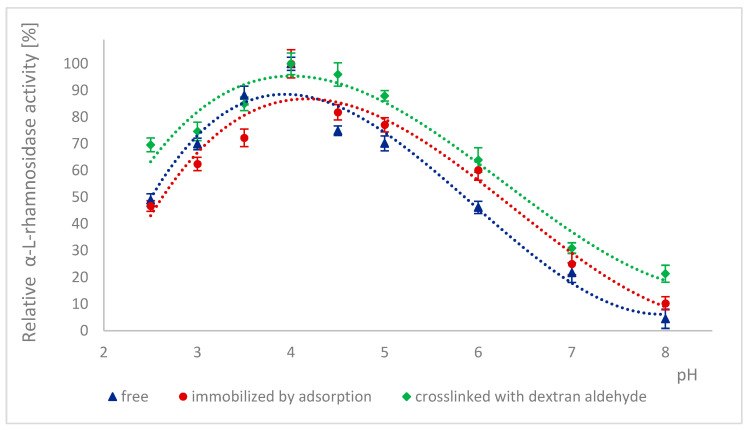
Effect of pH on the activity of α-L-rhamnosidase as a free, immobilized, and stabilized subunit of naringinase from *A. niger* KMS.

**Figure 2:**
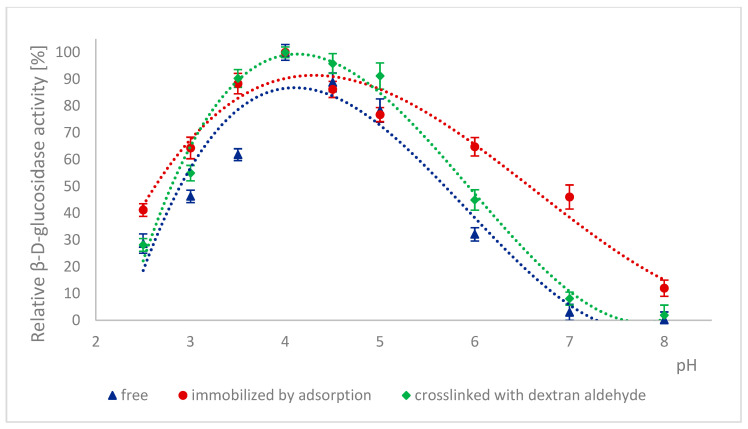
Effect of pH on the activity of β-D-glucosidase as a free, immobilized, and stabilized subunit of naringinase from *A. niger* KMS.

Because many enzymes exhibit high activity only within a narrow pH range, it is important to describe the effect of this factor on the enzymatic activity of α-L-rhamnosidase and β-D-glucosidase from free, immobilized, and stabilized naringinase. The effect of pH on the activity of free, immobilized, and stabilized naringinase subunits, α-L-rhamnosidase and β-D-glucosidase, is presented in Figure 1 and Figure 2. Changes in enzyme activity were studied in the pH range 2.5–8.0 at a temperature of 50 °C.

100% activity for free, immobilized and stabilized α-L-rhamnosidase was defined as the activity at pH 4.0 which was 887.17 ± 33 μmol·min^−1^·g^−1^, 4.82 ±0.12 μmol·min^−1^·g^−1^ and 5.09 ± 0.13 μmol·min^−1^·g^−1^, respectively. 100% activity for free, immobilized and stabilized β-D-glucosidase was defined as the activity at pH 4.0 which was 273.75 ± 13 μmol·min^−1^·g^−1^, 2.57± 0.06 μmol·min^−1^·g^−1^ and 3.02 ± 0.08 μmol·min^−1^·g^−1^, respectively.

The activity of all tested forms of α-L-rhamnosidase and β-D-glucosidase was highest at pH 4.0, which corresponded to the optimal pH for the activity of the free and immobilized on magnetic polysaccharide carrier naringinase from *A. niger* KMS complex [21]. Immobilization followed by stabilization of naringinase did not affect the optimal pH of its subunits, α-L-rhamnosidase and β-D-glucosidase.

Almost across the entire pH range analyzed, i.e., from 2.5 to 8.0, α-L-rhamnosidase from stabilized naringinase exhibits higher activity than the free and immobilized form of the biocatalyst under investigation. The activity of β-D-glucosidase from naringinase stabilized with dextran aldehyde was highest in the pH range from 3.5 to 5. Above pH 5, the highest activity was exhibited by β-D-glucosidase immobilized by adsorption. The high activity of the crosslinked α-L-rhamnosidase and β-D-glucosidase at low pH indicates the possibility of using such an immobilized enzyme in acidic medium as citrus juice.

The highest activity of both subunits at pH 4.0 is similar to the optimal pH of enzymes derived from *Aspergillus. moulds* and characterized by other authors: α-L-rhamnosidase [7,45,46,47] and β-D-glucosidase [7,46,48,49]. α-L-rhamnosidase from *A. terreus* [47], *A. kawachii* [45], *A. aculeatus* JMUdb058 [7], and *A. niger* BCC [46] showed the highest activity at pH 4.0. β-D-glucosidase from *A. aculeatus* JMUdb058 [7], *A. niger* BCC [46], and *Aspergillus aculeatus/Aspergillus niger* [49] showed the highest activity also at pH 4.0. Naringinase and its subunits from *A. aculeatus/A. niger* (NS 33117) also showed the highest activity at pH 4.0 [20,38].

Slightly different results were obtained by other authors for α-L-rhamnosidase and β-D-glucosidase from *A. oryzae* 11250, whose activity dependence on the reaction pH was similar. However, the optimum was reached at pH 5.0 [50]. Roitner et al. [51] showed that the activity of α-L-rhamnosidase from naringinase derived from *A. niger* was slightly dependent on pH in the range from 3 to 7, and β-D-glucosidase had a clear activity maximum at pH 5.

There is little information in the literature on the effect of immobilization on the activity of naringinase subunits depending on the pH of the reaction medium. Covalent binding of naringinase from *A. aculeatus/A. niger* (NS 33117) to butyl-glyoxyl agarose resulted in high activity of both subunits at pH 3–6 [49]. In contrast, in the case of immobilization on octyl-glyoxyl agarose, only α-L-rhamnosidase showed high activity [49]. The immobilization on glyoxyl-agarose of the aminated naringinase complex from *A. aculeatus/A. niger* (NS 33117) modified the pH profile of the enzyme and its subunits without changing the optimal pH (4.0). Only slight differences in the pH profile were observed after naringinase amination, particularly at pH above 4.0 [38].

### 2.4. Effect of Incubation in Buffers with Different pH on the Activity of Naringinase Subunits

**Figure 3:**
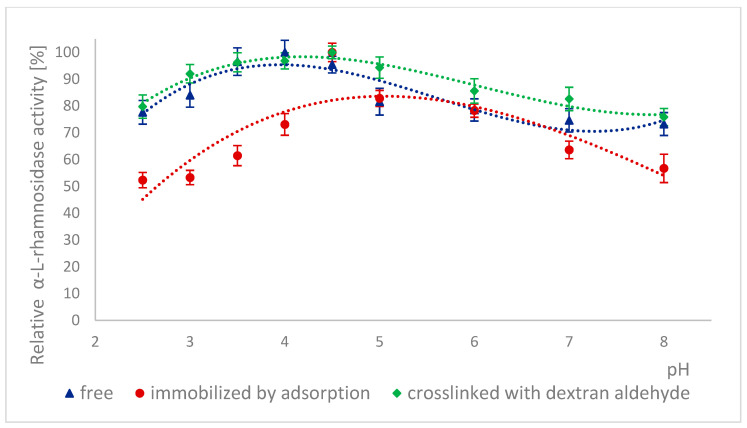
Stability of α-L-rhamnosidase as a free, immobilized, and stabilized subunit of naringinase from *A. niger* KMS after 15 h of incubation in buffers with different pH.

**Figure 4:**
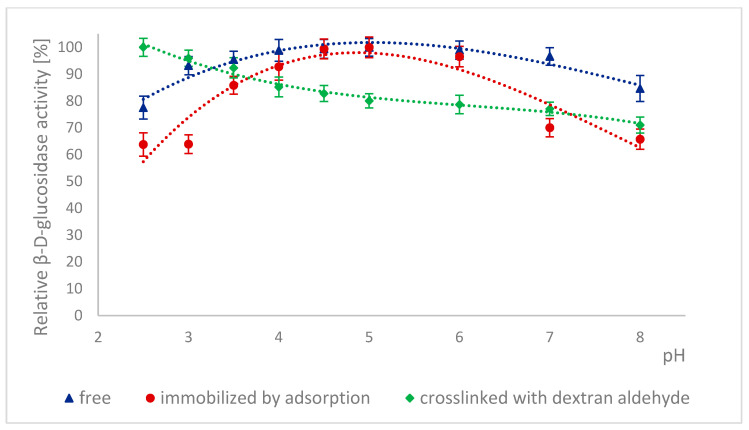
Stability of β-D-glucosidase as a free, immobilized, and stabilized subunit of naringinase from *A. niger* KMS after 15 h of incubation in buffers with different pH.

The high stability of naringinase subunits at different pH is advantageous for biotechnological processes in the food industry. The stability of α-L-rhamnosidase and β-D-glucosidase as subunits of free, immobilized, and stabilized naringinase was tested at different pH ranging from 2.5 to 8.0 (Figure 3 and Figure 4).

The free and cross-linked α-L-rhamnosidase pH stability profiles were similar (Figure 3). 100% activity for free α-L-rhamnosidase was defined as the activity at pH 4, which was 887.17 ± 33 μmol·min^−1^·g^−1^. 100% activity for immobilized and stabilized α-L-rhamnosidase was defined as the activity at pH 4.5, which was, respectively, 6.59 ± 0.16 μmol·min^−1^·g^−1^ and 5.26 ± 0.06 μmol·min^−1^·g^−1^.

Both forms reached maximum stability at pH 4.5. The immobilized enzyme also showed the highest stability at pH 4.5, but its stability profile differed. After incubation at pH levels lower and higher than optimal, the activity of this form of α-L-rhamnosidase decreased by approximately 20% at pH 5 and by approximately 27% at pH 4.

The pH stability profiles are presented in Figure 4. 100% activity for free β-D-glucosidase was defined as the activity at pH 5, which was 276.84 ± 13.15 μmol·min^−1^·g^−1^. 100% activity for immobilized β-D-glucosidase was defined as the activity at pH 5, which was 2.77 ± 0.065 μmol·min^−1^·g^−1^. 100% activity for stabilized β-D-glucosidase was defined as the activity at pH 2.5, which was 3.54 ± 0.094 μmol·min^−1^·g^−1^. For free and immobilized β-D-glucosidase, the pH stability profiles were similar, although at pH below 4.0 and above 6.0, the immobilized form lost activity more rapidly (Figure 4). It could be because immobilization changed the enzyme’s conformation, particularly in the active site, making it more sensitive to hydrogen and hydroxide ions. This conformational change was likely further enhanced by additional cross-links of the immobilized β-D-glucosidase, which led to a decrease in the activity of this form with increasing pH of the enzyme incubation environment.

Similar pH stability results were obtained by Chen et al. [7]. In this case, α-L-rhamnosidase from *A. aculeatus* JMUdb058 was stable at pH 3.0 to 8.0. At the same time, β-D-glucosidase was stable at pH 3.0 to 6.0. Slightly different results were obtained by Gallego et al. [47], and α-L-rhamnosidase from *A. terreus* was most stable at pH 4.0–6.0. However, Manzanares et al. [52] showed that α-L-rhamnosidase from *A. aculeatus* was stable at pH 3.0–5.0. The differences in the pH stability profile are probably because the authors used enzymes obtained from other *Aspergillus* species.

Low pH stability means that naringinase subunits, which were immobilized and cross-linked with dextran aldehyde, can be used to process acidic raw materials such as wines and citrus juices.

### 2.5. Effect of Temperature on the Activity of Naringinase Subunits

**Figure 5:**
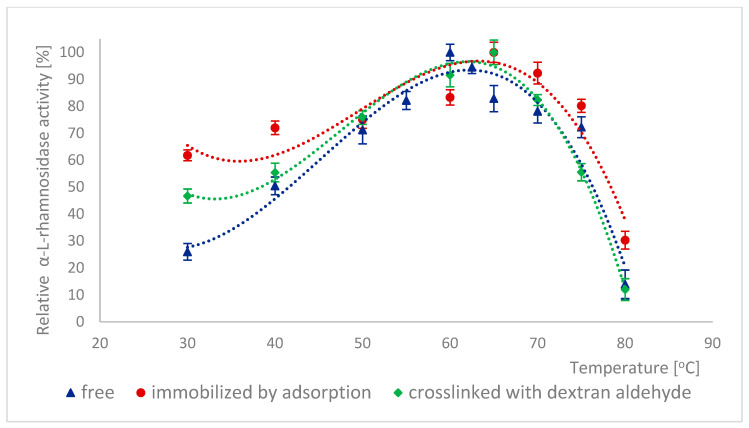
Effect of temperature on the activity of α-L-rhamnosidase as a subunit of free, immobilized, and stabilized naringinase from *A. niger* KMS.

**Figure 6:**
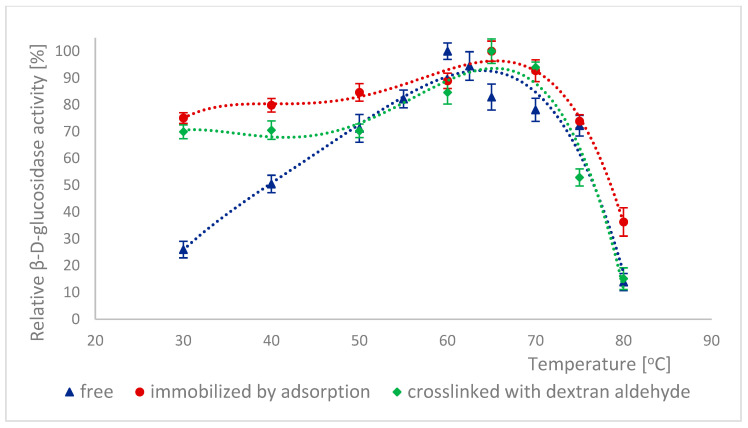
Effect of temperature on the activity of β-D-glucosidase as a subunit of free, immobilized, and stabilized naringinase from *A. niger* KMS.

Thermal inactivation of enzymes is often a significant problem in biotechnological processes. The effect of temperature on the activity of different forms of α-L-rhamnosidase is shown in Figure 5. 100% activity for free α-L-rhamnosidase was defined as the activity at 60 °C, which was 124.61 ± 6.35 μmol·min^−1^·g^−1^. 100% activity for immobilized and stabilized α-L-rhamnosidase was defined as the activity at 65 °C, which was, respectively, 6.41 ± 0.16 μmol·min^−1^·g^−1^ and 6.71 ± 0.08 μmol·min^−1^·g^−1^. For β-D-glucosidase, such relationships are shown in Figure 6. 100% activity for free β-D-glucosidase was defined as the activity at 60 °C, which was 608.88 ± 28.91 μmol·min^−1^·g^−1^. 100% activity for immobilized and stabilized α-L-rhamnosidase was defined as the activity at 65 °C, which was 3.04 ± 0.07 μmol·min^−1^·g^−1^ and 4.30 ± 0.11 μmol·min^−1^·g^−1^ respectively. In many cases, immobilization reduces the temperature sensitivity of biocatalysts. The method of immobilization and cross-linking of α-L-rhamnosidase and β-D-glucosidase with dextran aldehyde increased the temperature at which these enzymes achieve their highest activity by 5 °C compared to their free forms (Figure 5 and Figure 6). Furthermore, the activity of both immobilized enzyme forms also increased in the temperature range of 30–45 °C. Changes in their conformation likely caused the modification of the activity profiles of α-L-rhamnosidase and β-D-glucosidase due to immobilization and cross-linking. This change in the optimal temperature of the enzymes increases their potential for use in biotechnological processes. The differences in activity at optimal temperatures of free and immobilized subunits were statistically significant.

A similar optimal temperature (60 °C) in terms of activity was shown by α-L-rhamnosidase and β-D-glucosidase from *A. aculeatus*/*A. niger* (NS 3311) [38]. The optimal temperature remained unchanged after immobilizing the enzymes on polydopamine-coated magnetic iron oxide nanoparticles. In turn, the free naringinase complex from *A. niger* BCC 25166 and its subunits also showed maximum activity at 60 °C [46].

For α-L-rhamnosidase and β-D-glucosidase from other *Aspergillus* species, researchers determined different temperatures at which these enzymes achieved their highest activity. Naringinase subunits from *A. oryzae* 11250 were most active at 45 °C [50]. In turn, α-L-rhamnosidase and β-D-glucosidase from *A. aculeatus* JMUdb058 showed maximum activity at 50 °C [7] and from *A. terreus* also at 50 °C [53]. The optimal temperature for naringinase subunits from *A. aculeatus*/*A. niger* (NS 33117) varied depending on the substrate used, being 45 °C for p-nitrophenyl-β-D-glucoside (pNPG) and 60 °C for p-nitrophenyl-α-L-rhamnopyranoside (pNPR) [20]

Other authors also observed changes in the temperature at which naringinase subunits achieved maximum activity following immobilization. Muñoz et al. [49] found that immobilization on heterofunctional supports with glyoxyl groups increased the optimal temperature for α-L-rhamnosidase from *A. aculeatus*/*A. niger* (NS 33117) activity, whereas no changes were observed for β-D-glucosidase. A similar effect was observed by Urrutia et al. [20] for aminated naringinase from *A. aculeatus*/*A. niger* (NS 33117) bound to a glyoxyl-agarose support, where the optimal temperature for α-L-rhamnosidase shifted from 40 to 60 °C.

### 2.6. Thermal Stability of Naringinase Subunits

**Figure 7:**
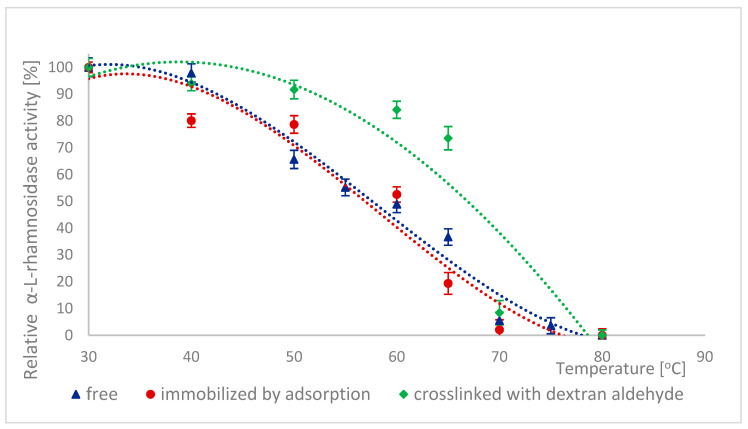
Thermal stability of α-L-rhamnosidase as a free, immobilized, and stabilized subunit of naringinase from *A. niger* KMS.

**Figure 8:**
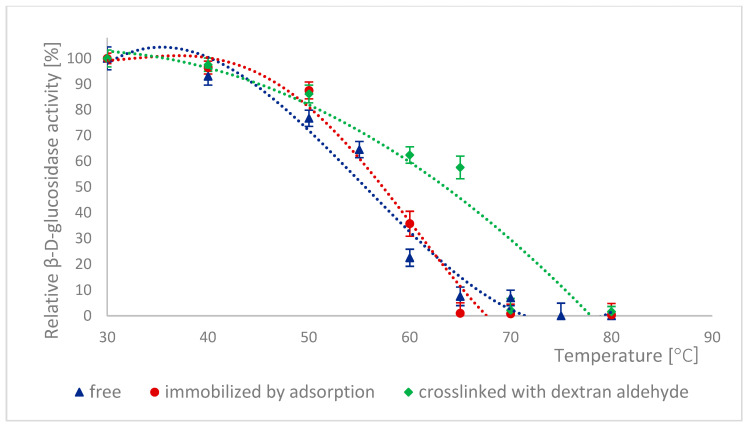
Thermal stability of β-D-glucosidase as a free, immobilized, and stabilized subunit of naringinase from *A. niger* KMS.

As a protein, naringinase is sensitive to temperature; hence, improving its thermal stability by immobilizing the enzyme is desirable. The thermal stability of naringinase subunits, i.e., α-L-rhamnosidase and β-D-glucosidase, was determined by incubating naringinase in acetate buffer (pH 4.0) at temperatures ranging from 30 to 80 °C, followed by the determination of the activity of both enzymes. Figure 7 and Figure 8 show the changes in the activity of both enzymes as a function of their incubation temperature. 100% activity for free, immobilized and stabilized α-L-rhamnosidase was defined as the activity at 30 °C, which was 130.77 ± 36 μmol·min^−1^·g^−1^, 6.24 ± 0.16 μmol·min^−1^·g^−1^ and 5.90 ± 0.07 μmol·min^−1^·g^−1^, respectively. For β-D-glucosidase, 100% activity for free, immobilized, and stabilized β-D-glucosidase was defined as the activity at 30 °C, which was 417.03 ± 19.80 μmol·min^−1^·mg^−1^ 3.27 ± 0.076 μmol·min^−1^·g^−1^ and 3.29 ± 0.087 μmol·min^−1^·g^−1^, respectively.

α-L-rhamnosidase was the most stable among the tested enzyme forms, a subunit of immobilized and dextran aldehyde-stabilized naringinase. It is worth noting that the rate of decrease in its activity increased only after exceeding a temperature of 60 °C. α-L-rhamnosidase from naringinase bound with dextran aldehyde was therefore thermally stable at temperatures between 30 and 60 °C.

In the 20–50 °C temperature range, no significant changes in thermal stability were observed between the different forms of this enzyme. Dextran aldehyde cross-linking of immobilized β-D-glucosidase increased thermal stability above an incubation temperature of 50 °C compared to the other forms of this enzyme. Cross-linking of immobilized α-L-rhamnosidase and β-D-glucosidase increased the thermal stability of both subunits, likely due to stabilization of the altered spatial structure of the enzymes.

The differences in the stability of free and immobilized α-L-rhamnosidase base at 30 °C were not statistically significant. For β-D-glucosidase, the differences were not statistically significant at 30–40 °C. In the case of free and immobilized α-L-rhamnosidase, the differences in thermal stability at temperatures above 70 °C were not statistically significant. For β-D-glucosidase, they were not statistically significant above 65 °C.

The high activity of both subunits, in free and immobilized form, at low temperatures makes the process particularly suitable for the hydrolysis of flavonoids in juices or wine, as it helps preserve thermolabile components (such as vitamin C and β-carotene) and maintain the desirable sensory qualities of the final product.

The stability of both naringinase subunits determines the thermal stability of the entire enzyme complex. The naringinase complex from *A. aculeatus* JMUdb058 and its subunits, α-L-rhamnosidase and β-D-glucosidase, also exhibited similar temperature stability ranges below 50 °C [7]. Kimmins et al. [38] also observed an increase in the stability of the naringinase complex from *A. aculeatus*/*A. niger* (NS 33117) on polydopamine-coated magnetic iron oxide nanoparticles at 50 °C. After 120 min of incubation, the immobilized α-L-rhamnosidase retained more than 50% of its initial activity, whereas the free form showed only about 30%. In the case of β-D-glucosidase, immobilization allowed maintaining about 20% of the initial activity, while the free enzyme completely lost its activity.

### 2.7. Determination of the Kinetic Parameters of Naringinase Subunits

Immobilization often changes the properties of the immobilized enzyme, particularly parameters such as the Michaelis-Menten constant (K_M_) and the maximum enzyme reaction rate (V_max_).

**Figure 9:**
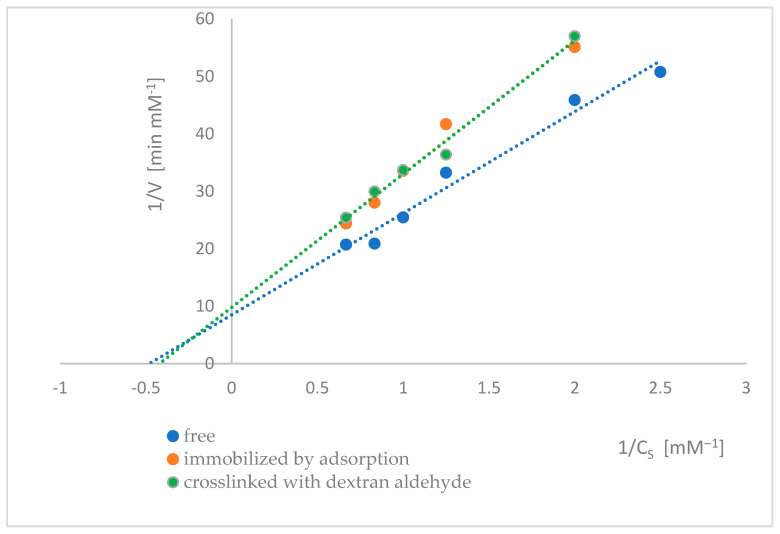
Lineweaver–Burk plots of α-L-rhamnosidase as a free, immobilized, and stabilized subunit of naringinase from *A. niger* KMS.

**Figure 10:**
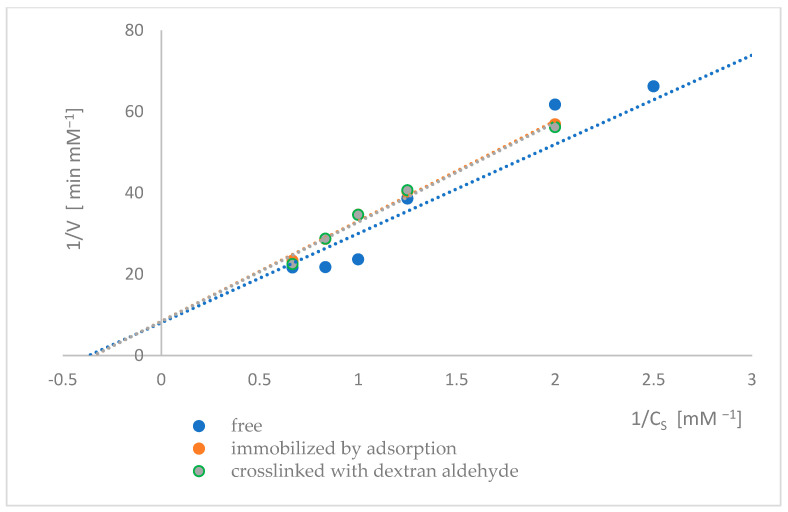
Lineweaver–Burk plots of β-D-glucosidase as a free, immobilized, and stabilized subunit of naringinase from *A. niger* KMS.

The kinetic parameters of naringinase subunits, i.e., α-L-rhamnosidase and β-D-glucosidase, were determined from the dependence of the initial reaction rate on the substrate concentration. The Lineweaver-Burk method determined the K_M_ and V_max_ (Figure 9 and Figure 10). The values of the kinetic parameters of α-L-rhamnosidase and β-D-glucosidase of free, immobilized, and dextran aldehyde-bound naringinase are presented in Table 2 and Table 3.

The K_M_ of α-L-rhamnosidase from free naringinase was slightly lower than that of β-D-glucosidase. It means that α-L-rhamnosidase has a higher affinity for pNPR than β-D-glucosidase has for pNPG. Other authors obtained similar results for naringinase subunits, where the K_M_ of β-D-glucosidase was higher than that of α-L-rhamnosidase. Ni et al. [6] found that the α-L-rhamnosidase from *A. niger* DB056 has a lower V_max_ (1.51 μM·mL^−1^) than the β-D-glucosidase (9.63 μM·mL^−1^), implying the hydrolysis of naringin to prunin was the velocity-limiting step of the naringin degradation.

The K_M_ of α-L-rhamnosidase from *A. oryzae* 11250 toward pNPR was estimated to be 0.63 mM, which is lower than that of β-D-glucosidase for pNPR (2.8 mM) [50]. The K_M_ of α-L-rhamnosidase from *A. sojae* toward pNPR was estimated to be 1.79 mM, which is lower than that of β-D-glucosidase for pNPR (239 mM) [54]. α-L-Rhamnosidase from *A. niger* DB056 had a K_M_ of 0.28 mM, whereas β-D-glucosidase had a K_M_ of 0.39 mM [6]. α-L- Rhamnosidase from *A. terreus* had a K_M_ of 0.52 mM, while for β-D-glucosidase, the K_M_ was 1.89 mM [53]. The differences may have resulted from species differences and the culture conditions of the microorganisms used to obtain naringinase.

The results presented in Table 2 showed that the immobilization of naringinase increased the Michaelis-Menten constant of α-L-rhamnosidase compared to the free enzyme. Further stabilization of the binding of naringinase with dextran aldehyde did not significantly affect the Michaelis-Menten constant, suggesting that the affinity toward the substrate was reduced when the enzyme was immobilized. A lower maximum reaction rate was accompanied an increase in the apparent Michaelis constant (decreased enzyme affinity for the substrate). This is likely related to conformational changes in the naringinase subunits, the hydrodynamic conditions of the environment, and the electrochemical conditions of both the carrier and the substrate.

As a result of immobilization of the tested biocatalyst, the maximum reaction rate of α-L-rhamnosidase decreased slightly. The binding of immobilized naringinase with dextran aldehyde did not significantly affect the maximum reaction rate of α-L-rhamnosidase compared to the enzyme immobilized by adsorption.

The Michaelis-Menten constant of immobilized β-D-glucosidase was slightly higher than that of the free enzyme. Stabilizing immobilized naringinase with dextran aldehyde did not significantly affect the K_M_ compared to the enzyme immobilized by adsorption, suggesting that the affinity toward the substrate was reduced when the enzyme was immobilized.

Immobilization of naringinase on a magnetic carrier resulted in a significant reduction in the maximum reaction rate of β-D-glucosidase compared to the free enzyme. Further binding of naringin with dextran aldehyde resulted in a slight decrease in the maximum reaction rate.

As a result of the immobilization and stabilization of naringinase, α-L-rhamnosidase showed a lower V_max_ than β-D-glucosidase, suggesting that α-L-rhamnosidase is the main factor inhibiting the rate of naringin hydrolysis by naringinase.

As a result of immobilization, the V_max_ of both subunits decreased, indicating a lower catalytic efficiency of the immobilized enzyme. This may be caused by the limited accessibility of the enzyme to pNPR/pNPG and the diffusion of p-nitrophenol [30]. As a result of immobilization, the K_M_ of both subunits increased, thus decreasing the affinity of the enzymes for substrates. Nevertheless, higher K_M_ and lower V_max_ after immobilization are typical for immobilized biocatalysts [55]. Usually, this is due to diffusion limitations in the microenvironment and steric constraints that reduce substrate accessibility and enzyme flexibility [56].

There are no reports in the literature on the effect of naringinase immobilization on the kinetic parameters of its subunits. Only the effect of immobilization on individual enzymes, α-L-rhamnosidase and β-D-glucosidase, was studied. Similar results to ours were obtained in the study by Spagna et al. [56], where the K_M_ of α-L-rhamnosidase from *A. niger* immobilized on chitosan activated with glutaraldehyde doubled, and the V_max_ decreased twofold compared with the free enzyme. β-D-glucosidase from *A. niger* immobilized in alginate showed an apparent K_M_ higher and V_max_ lower than the free enzyme [57].

Slightly different results were obtained by immobilizing α-L-rhamnosidase from *A. niger* on a magnetic metal–organic framework. Compared with the free enzyme, immobilized α-L-rhamnosidase showed a relatively minor V_max_ and a lower *K*_M_ [30]. The authors explained this change in K_M_ because the active site was more accessible to the enzyme due to a conformational change after immobilization on a carrier with a high specific surface area.

In the literature, a different relationship was reported for naringinase subunits from *A. niger* DB056 [6], *A. terreus* [53], and *A. oryzae* 11250 [50]. The differences may have resulted from species differences and the culture conditions of the microorganisms used to obtain naringinase.

## 3. Materials and Methods

### 3.1. Materials

Naringinase from *A. niger* KMS, cultured under submerged conditions, was isolated by concentrating the post-culture fluid through ultrafiltration, followed by protein precipitation using acetone, yielding a solid preparation with an activity of 816 µmol·min^−1^·g^−1^. p-nitrophenyl-α-L-rhamnopyranoside (pNPR) and p-nitrophenyl-β-D-glucoside (pNPG) were purchased from Sigma-Aldrich (St. Louis, MO, USA). Polysaccharides and activators were used for enzyme immobilization: carob gum obtained from *Ceratonia siliqua* (Sigma-Aldrich, St. Louis, MO, USA) and a 50% aqueous solution of polyethyleneimine (Sigma-Aldrich, St. Louis, MO, USA).

### 3.2. Analytical Methods

#### 3.2.1. Determination of α-L-rhamnosidase and β-D-glucosidase Activity from Free Naringinase

The method described by Spagna et al. assessed the activity of α-L-rhamnosidase and β-D-glucosidase [56,58]. The method was based on spectrophotometric determination of the concentration of p-nitrophenol obtained in the decomposition reaction of p-nitrophenyl-α-rhamnopyranoside or p-nitrophenyl-α-glucopyranoside in reaction with α-L-rhamnosidase and β-D-glucosidase, respectively. The absorbance of the solutions was measured at a wavelength of λ = 410 nm. The absorbance was then converted to the concentration of p-nitrophenol using a regression equation characterizing the standard curve, prepared based on measurements of the absorbance of aqueous solutions of p-nitrophenol in the concentration range 0.01–0.20 mM. In the case of determining the activity of free naringinase subunits, 0.4 cm^3^ 2 mM solution of pNPR or pNPG was dissolved in 0.1 M acetate buffer (pH 4.0), and 0.01 cm^3^ of naringinase solution was added to 0.09 cm^3^ of water. The reaction was carried out for 5 or 10 min, after which 1 cm^3^ 1 M Na_2_CO_3_ was added to stop the reaction.

The α-L-rhamnosidase and β-D-glucosidase activity is expressed in µmol of pNPR or pNPG hydrolyzed for 1 min by 1 g of the enzyme preparation.

#### 3.2.2. Determination of α-L-rhamnosidase and β-D-glucosidase Activity from Immobilized and Stabilized Naringinase

To determine the activity of α-L-rhamnosidase and β-D-glucosidase of naringinase bound to a carrier, 0.09 cm^3^ of water and 0.4 cm^3^ of 2 mM solution of pNPR or pNPG dissolved in 0.1 M acetate buffer (pH 4.0) were added to 37.5 mg of carrier with immobilized enzyme. The reaction was carried out for 5 or 10 min, after which 1 cm^3^ 1 M Na_2_CO_3_ was added to stop the reaction.

The α-L-rhamnosidase and β-D-glucosidase activity is expressed in µmol of pNPR or pNPG hydrolyzed for 1 min by 1 g of the immobilized preparation.

### 3.3. Immobilization of Naringinase

Naringinase from *A. niger* KMS was adsorbed onto a magnetic carrier obtained from carob gum activated with polyethyleneimine, as described in our previous work. In order to strengthen the bond between the enzyme and the carrier, naringinase was additionally stabilized by cross-linking with dextran aldehyde [21].

Immobilization of naringinase from *A. niger* KMS: 7.5 cm^3^ of enzyme solution (50 mg·10 mL^−1^ 0.1 M phosphate buffer, pH 7.0) was added to 150 mg of carrier. The mixture was incubated at 27 °C for 4 h, shaking at a frequency of 150 rpm.

Cross-linking bound naringinase with dextran aldehyde: After completion of the immobilization stage, 10 cm^3^ of 20% dextran aldehyde solution was added to the mixture. Incubation was continued for another 20 h under the same conditions.

After the enzyme stabilization stage using dextran aldehyde, the enzyme-carrier complex was separated from the solution using a neodymium magnet and then rinsed several times with phosphate buffer (0.01 M, pH 7) and distilled water.

#### Immobilization Yield

The activity immobilization yield (Ya) was calculated from the following equation:

(1) $$Y_{a}=\frac{Xa}{{Xa}_{0}}\;\times 100\%,$$

where

Xa—the difference between the total activity of the enzyme used for immobilization and the activity of the immobilized enzyme;
Xa_0_—the total activity of the enzyme used for immobilization.
100% yield was defined as the total activity of naringinase (Xa_0_) immobilized.

### 3.4. Characteristics of Free, Immobilized and Stabilized Biocatalyst

#### 3.4.1. Effect of pH on the Activity of α-L-rhamnosidase and β-D-glucosidase

The effect of the active acidity of the reaction environment on the activity of α-L-rhamnosidase and β-D-glucosidase from free, immobilized, and stabilized naringinase was determined by analyzing the activity of the enzymes at different pH of the mixture. Briefly, 0.1 M McIlvaine buffer (pH 2.5–8.0) was used. The activity of the biocatalysts was tested at 50 °C. The activity of α-L-rhamnosidase and β-D-glucosidase was determined using the methods described in Section 3.2.1 and Section 3.2.2. The activity of α-L-rhamnosidase and β-D-glucosidase was determined.

#### 3.4.2. Effect of Incubation in Buffers with Different pH on the Activity of α-L-rhamnosidase, and β-D-glucosidase

The stability of free, immobilized, and crosslinked naringinase subunits depending on the pH of the environment was tested by incubating the enzymes for 15 h in buffers with different pH. Briefly, 0.1 M McIlvaine buffer (pH 2.5–8.0) was used. The activity of the biocatalysts was tested at 50 °C in a buffer with an optimal pH of 4.0. The activity of α-L-rhamnosidase and β-D-glucosidase was determined using the methods described in Section 3.2.1 and Section 3.2.2.

#### 3.4.3. Effect of Temperature on the Activity of α-L-rhamnosidase and β-D-glucosidase

The effect of temperature on changes in the enzymatic activity of α-L-rhamnosidase and β-D-glucosidase of free and immobilized naringinase was studied at temperatures ranging from 30 to 80 °C. The activity of the studied biocatalysts was determined in a 0.1 M acetate buffer with a pH of 4.0. The activity of α-L-rhamnosidase and β-D-glucosidase was determined using the methods described in Section 3.2.1 and Section 3.2.2.

#### 3.4.4. Thermal Stability of α-L-rhamnosidase and β-D-glucosidase

The thermal stability of α-L-rhamnosidase and β-D-glucosidase from free, immobilized, and stabilized naringinase was tested by incubating all enzyme forms at a specified temperature for 60 min. The incubation was carried out at temperatures ranging from 30 to 80 °C. After 60 min, the enzymes were cooled in an ice bath. After that, the remaining activity of the tested enzymes was determined at 50 °C in 0.1 M buffer with a pH of 4.0. The activity of α-L-rhamnosidase and β-D-glucosidase was determined using the methods described in Section 3.2.1 and Section 3.2.2.

#### 3.4.5. Determination of Kinetic Parameters

The kinetic parameters of naringinase subunits were studied in the decomposition reaction of p-nitrophenyl-α-rhamnopyranoside (pNPR) or p-nitrophenyl-β-glucopyranoside (pNPG) by α-L-rhamnosidase and β-D-glucosidase, respectively. The assay mixture (1.5 mL) was prepared with either 0.05 mg of free naringinase or 9 mg of immobilized enzyme. The reaction rate was determined at 50 °C in a 0.1 M buffer with a pH of 4.0 using substrate concentrations (pNPR or pNPG) in the reaction mixture equal to 0.4 mM, 0.5 mM, 0.8 mM, 1 mM, 1.5 mM. For each substrate concentration, four samples were prepared. In the first test, the product concentration was determined after 1 min of reaction; in the second test, after 2 min; in the third test, after 3 min; and finally, after 5 min. The initial reaction rate for each substrate concentration was calculated from the derivative of the product formation rate at the start of the reaction.

The maximum reaction rate (V_max_) and Michaelis constant (K_M_) were calculated using the Lineweaver–Burk method based on the dependence of the reaction rate on the substrate concentration:

(2) $$\frac{1}{\nu }=\frac{K_{M}}{\nu_{max}}*\frac{1}{C_{s}}+\frac{1}{\nu_{max}}$$

where

ν—enzyme reaction rate [mM·min^−1^];
ν_max_—maximum reaction rate [mM·min^−1^];
K_M_—Michaelis constant [mM];
C_s_—substrate concentration [mM].

### 3.5. Statistical Analysis

All determinations were performed in triplicate. All values are expressed as mean ± standard deviation of the three replicate experiments. STATISTICA v13.3 was used to evaluate the data.

## 4. Conclusions

The naringinase complex was adsorbed onto a magnetic polysaccharide carrier obtained from carob gum activated with polyethyleneimine. In order to strengthen the bond between the enzyme and the carrier, the complex was additionally stabilized by cross-linking with dextran aldehyde. This immobilization procedure was developed for naringinase and described in detail in an earlier article [21]. This approach increased the pH stability of α-L-rhamnosidase in the wide pH range of 3.0–7.0 and β-D-glucosidase at a pH below 3.0. It expands the possibilities of using the tested enzymes in an acidic environment typical of citrus juices, e.g., in the hydrolysis of naringin in grapefruit juice or hesperidin in orange juice. Immobilization and additional and subsequent stabilization of naringinase increased the activity of both subunits at low temperatures below 40 °C. At these temperatures and with short exposure times, the stability of thermolabile components of citrus fruit juices is higher, which does not cause any noticeable changes in the sensory and nutritional quality of the products. The stability of α-L-rhamnosidase and β-D-glucosidase increased at temperatures above 50 °C, broadening the potential application of these enzymes in the pharmaceutical industry, e.g., in producing valuable prunin.

The enzymatic characteristics of naringinase subunits, i.e., α-L-rhamnosidase and β-D-glucosidase, allow their range of activity, optimal pH, and temperature to be determined, which is essential for their practical use. Thanks to the targeted deglycosylation of flavonoids, including hesperidin, naringenin, and rutin, their bioavailability can be increased. For this purpose, it is necessary to preserve the activity of only α-L-rhamnosidase from the complex naringinase enzyme and to inhibit the second enzyme, β-D-glucosidase, using specific inhibitors or reaction conditions that ensure the selective activity of the enzymatic subunits of naringinase. An analysis of the literature clearly shows that knowledge in this area is limited and needs to be supplemented.

## Figures and Tables

**Table 1:** Activity of α-L-rhamnosidase and β-D-glucosidase as free, immobilized, and cross-linked subunits of naringinase.

Enzyme	Free	Adsorbed	Adsorbed and Crosslinked
	µmol·min^−1^ g^−1^	µmol·min^−1^·g^−1^ of Carrier	µmol·min^−1^·g^−1^ of Carrier
α-L-rhamnosidase	887.17 ± 33	4.82 ±0.12	5.09 ± 0.13
β-D-glucosidase	273.75 ± 13	2.57 ± 0.06	3.02 ± 0.08

**Table 2:** The values of the kinetic parameters of α-L-rhamnosidase as a free, immobilized, and stabilized subunit of naringinase from *A. niger* KMS.

Type of Enzyme	V_max_ [mM^·^min^−1^]	K_M_ [mM]
Free α-L-rhamnosidase	0.1176 ± 0.0016 ^(a)^	2.0803 ± 0.0355 ^(c)^
Adsorbed α-L-rhamnosidase	0.1019 ± 0.0044 ^(b)^	2.3698 ± 0.1288 ^(d)^
Adsorbed α-L-rhamnosidase crosslinked with dextran aldehyde	0.1021 ± 0.0053 ^(b)^	2.3678 ± 0.1359 ^(d)^

Different letters indicate statistically significant differences at the *p* < 0.05 level.

**Table 3:** The values of the kinetic parameters of β-D-glucosidase as a free, immobilized, and stabilized subunit of naringinase from *A. niger* KMS.

Type of Enzyme	V_max_ [mM^·^min^−1^]	K_M_ [mM]
Free β-D-glucosidase	0.1240 ± 0.0032 ^(a)^	2.7213 ± 0.0780 ^(c)^
Adsorbed β-D-glucosidase	0.1188 ± 0.0026 ^(b)^	2.9280 ± 0.0780 ^(d)^
Adsorbed β-D-glucosidase crosslinked with dextran aldehyde	0.1195 ± 0.0019 ^(b)^	2.9277 ± 0.0565 ^(d)^

Different letters indicate statistically significant differences at the *p* < 0.05 level.

## Data Availability

Data will be made available on request. Public data archive is not available.

## Abbreviations

The following abbreviations are used in this manuscript:

Hes-7-G	hesperetin 7-O-glucoside
pNPR	4-nitrophenyl α-L-rhamnopyranoside
pNPG	4-nitrophenyl β-D-glucopyranoside
νmax	maximum reaction rate
K_M_	Michaelis constant

## References

[1] Puri M. (2012). Updates on naringinase: Structural and biotechnological aspects. Appl. Microbiol. Biotechnol..

[2] Golgeri M.D.B., Mulla S.I., Bagewadi Z.K., Faniband B., Mishra P., Bankole P.O., Sharma S., Américo-Pinheiro J.H.P., Bharagava R.N., Romanholo Ferreira L.F. (2025). Microbial naringinase: From microbial source to its current applications in various fields. Biologia.

[3] Cui Z., Maruyama Y., Mikami B., Hashimoto W., Murata K. (2007). Crystal Structure of Glycoside Hydrolase Family 78 α-L-Rhamnosidase from *Bacillus* sp. GL1. J. Mol. Biol..

[4] Zhu Y., Jia H., Xi M., Xu L., Wu S., Li X. (2017). Purification and characterization of a naringinase from a newly isolated strain of *Bacillus amyloliquefaciens* 11568 suitable for the transformation of flavonoids. Food Chem..

[5] Slámová K., Kapešová J., Valentová K. (2018). “Sweet Flavonoids”: Glycosidase-Catalyzed Modifications. Int. J. Mol. Sci..

[6] Ni H., Xiao A.-F., Wang Y.Q., Chen F., Cai H.-N., Su W.-J. (2013). Development and evaluation of an HPLC method for accurate determinations of enzyme activities of naringinase complex. J. Agric. Food Chem..

[7] Chen Y., Ni H., Chen F., Cai H., Li L., Su W. (2013). Purification and characterization of a naringinase from *Aspergillus aculeatus* JMUdb058. J. Agric. Food Chem..

[8] Ni H., Chen F., Cai H., Xiao A., You Q., Lu Y. (2012). Characterization and preparation of *Aspergillus niger* naringinase for debittering citrus juice. J. Food Sci..

[9] Soria F., Ellenrieder G., Grasselli M., Navarro Del Cañizo A.A., Cascone O. (2004). Fractionation of the naringinase complex from *Aspergillus terreus* by dye affinity chromatography. Biotechnol. Lett..

[10] Bodakowska-Boczniewicz J., Garncarek Z. (2025). Use of Naringinase to Modify the Sensory Quality of Foods and Increase the Bioavailability of Flavonoids: A Systematic Review. Molecules.

[11] Ribeiro M.H. (2011). Naringinases: Occurrence, characteristics, and applications. Appl. Microbiol. Biotechnol..

[12] Li B., Wu B., Hou X., Ding G. (2025). Substrate Selectivities of GH78 α -L-Rhamnosidases from Human Gut Bacteria on Dietary Flavonoid Glycosides. Molecules.

[13] RoitNer M., Schalkhammer T., Pittner F. (1984). Preparation of prunin with the help of immobilized naringinase pretreated with alkaline buffer. Appl. Biochem. Biotechnol..

[14] Rajendran D.S., Chidambaram A., Kumar P.S., Venkataraman S., Muthusamy S., Vo D.-V.N., Rangasamy G., Vaithyanathan V.K., Vaidyanathan V.K. (2023). Three-phase partitioning for the separation of proteins, enzymes, biopolymers, oils and pigments: A review. Environ. Chem. Lett..

[15] Mazzaferro L., Piñuel L., Minig M., Breccia J.D. (2010). Extracellular monoenzyme deglycosylation system of 7-O-linked X avonoid-rutinosides and its disaccharide transglycosylation activity from *Stilbella fimetaria*. Arch. Microbiol..

[16] Lee Y.S., Huh J.Y., Nam S.H., Moon S.K., Lee S.B. (2012). Enzymatic bioconversion of citrus hesperidin by *Aspergillus sojae* naringinase: Enhanced solubility of hesperetin-7-O-glucoside with in vitro inhibition of human intestinal maltase, HMG-CoA reductase, and growth of *Helicobacter pylori*. Food Chem..

[17] Zou Y., Xin X., Xu H., Yuan H., Li X., Yu Y., Zhao G. (2020). Highly efficient bioconversion of flavonoid glycosides from citrus-processing wastes in solvent-buffer systems. Green Chem..

[18] Vila-Real H., Alfaia A.J., Bronze M.R., Calado A.R.T., Ribeiro M.H.L. (2011). Enzymatic Synthesis of the Flavone Glucosides, Prunin and Isoquercetin, and the Aglycones, Naringenin and Quercetin, with Selective α-L-Rhamnosidase and β-D-Glucosidase Activities of Naringinase. Enzym. Res..

[19] Li L.J., Liu X.Q., Du X.P., Wu L., Jiang Z.D., Ni H., Li Q.B., Chen F. (2020). Preparation of isoquercitrin by biotransformation of rutin using α-L-rhamnosidase from *Aspergillus niger* JMU-TS528 and HSCCC purification. Prep. Biochem. Biotechnol..

[20] Urrutia P., Arrieta R., Torres C., Guerrero C., Wilson L. (2024). Amination of naringinase to improve citrus juice debittering using a catalyst immobilized on glyoxyl-agarose. Food Chem..

[21] Bodakowska-Boczniewicz J., Garncarek Z. (2020). Immobilization of Naringinase from *Aspergillus niger* on a Magnetic Polysaccharide Carrier. Molecules.

[22] Bilal M., Iqbal H.M.N. (2019). Chemical, physical, and biological coordination: An interplay between materials and enzymes as potential platforms for immobilization. Coord. Chem. Rev..

[23] Ribeiro I.A.C., Ribeiro M.H.L. (2008). Kinetic modelling of naringin hydrolysis using a bitter sweet alfa-rhamnopyranosidase immobilized in k-carrageenan. J. Mol. Catal. B Enzym..

[24] Bodakowska-Boczniewicz J., Garncarek Z. (2019). Immobilizacja naringinazy z *Penicillium decumbens* na magnetycznych polisacharydowych nośnikach. Pr. Nauk. Uniw. Ekon. We Wrocławiu.

[25] Ferreira L., Afonso C., Vila-real H., Alfaia A. (2008). Evaluation of the Effect of High Pressure on Naringin Hydrolysis in Grapefruit Juice with Naringinase Immobilised in Calcium Alginate Beads. Food Technol. Biotechnol..

[26] Norouzian D., Hosseinzadeh A., Inanlou D.N., Moazami N. (1999). Various techniques used to immobilize naringinase produced by *Penicillium decombens* PTCC 5248. World, J. Microbiol. Biotechnol..

[27] Awad G.E.A., Abd El Aty A.A., Shehata A.N., Hassan M.E., Elnashar M.M. (2016). Covalent immobilization of microbial naringinase using novel thermally stable biopolymer for hydrolysis of naringin. 3 Biotech.

[28] Garzón-Alonso K.E., Bohorquez-Peña M.J., Vargas-Suaza B., Rendón-Londoño J.C., Mesa M., Franco-Tobón Y.N., Martínez-Galán J.P. (2025). Hydrolysis of flavanones from orange peel and evaluation of anticancer potential using naringinase immobilized on corn cob powder. Food Biosci..

[29] Serra I., Serra C.D., Rocchietti S., Ubiali D., Terreni M. (2011). Stabilization of thymidine phosphorylase from *Escherichia coli* by immobilization and post immobilization techniques. Enzym. Microb. Technol..

[30] Wang D., Zheng P., Chen P., Wu D. (2021). Immobilization of alpha-L-rhamnosidase on a magnetic metal-organic framework to effectively improve its reusability in the hydrolysis of rutin. Bioresour. Technol..

[31] Wan W., Xia N., Zhu S., Liu Q., Gao Y. (2020). A Novel and High-Effective Biosynthesis Pathway of Hesperetin-7-O-Glucoside Based on the Construction of Immobilized Rhamnosidase Reaction Platform. Front. Bioeng. Biotechnol..

[32] Wei B., Liu F., Liu X., Cheng L., Yuan Q., Gao H., Liang H. (2022). Enhancing stability and by-product tolerance of β-glucuronidase based on magnetic cross-linked enzyme aggregates. Colloids Surf. B Biointerfaces.

[33] Hadadi M., Habibi A. (2024). Development of Magnetic Immobilized Cellulase Biocatalysts for Saccharification of Paper Waste. Catal. Lett..

[34] He G., Liu H., Yang C., Hu K., Zhai X., Fang B., Liu K., Zulekha, Li D. (2024). A comparison of dual-enzyme immobilization by magnetic nanoparticles and magnetic enzyme aggregates for cascade enzyme reactions. Biochem. Eng. J..

[35] Cavalcante A.L.G., Dari D.N., Aires F.I.d.S., de Castro E.C., dos Santos K.M., dos Santos J.C.S. (2024). Advancements in enzyme immobilization on magnetic nanomaterials: Toward sustainable industrial applications. RSC Adv..

[36] Yu C., Li Q., Tian J., Zhan H., Zheng X., Wang S., Sun X., Sun X. (2021). A facile preparation of immobilized naringinase on polyethyleneimine-modified Fe_3_O_4_ magnetic nanomaterials with high activity. RSC Adv..

[37] Ladole M.R., Pokale P.B., Varude V.R., Belokar P.G., Pandit A.B. (2021). One pot clarification and debittering of grapefruit juice using co-immobilized enzymes@chitosanMNPs. Int. J. Biol. Macromol..

[38] Kimmins S.D., Henríquez A., Torres C., Wilson L., Flores M., Pio E., Jullian D., Urbano B., Braun-Galleani S., Ottone C. (2024). Immobilization of Naringinase onto Polydopamine-Coated Magnetic Iron Oxide Nanoparticles for Juice Debittering Applications. Polymers.

[39] Luo J., Li Q., Sun X., Tian J., Fei X., Shi F., Zhang N., Liu X. (2019). The study of the characteristics and hydrolysis properties of naringinase immobilized by porous silica material. RSC Adv..

[40] Liu R., Chen D., Fu H., Lv P., Zhang D., He Y. (2017). A facile preparation process of magnetic aldehyde-functionalized Ni0.5Zn0.5Fe_2_O_4_@SiO_2_ nanocomposites for immobilization of Penicillin G Acylase (PGA). J. Nanosci. Nanotechnol..

[41] Busto M.D., Meza V., Ortega N., Perez-Mateos M. (2007). Immobilization of naringinase from *Aspergillus niger* CECT 2088 in poly(vinyl alcohol) cryogels for the debittering of juices. Food Chem..

[42] Puri M., Marwaha S.S., Kothari R.M. (1996). Studies on the applicability of alginate-entrapped naringinase for the debittering of kinnow juice. Enzym. Microb. Technol..

[43] Vila-Real H., Alfaia A.J., Rosa M.E., Calado A.R., Ribeiro M.H.L. (2010). An innovative sol-gel naringinase bioencapsulation process for glycosides hydrolysis. Process Biochem..

[44] Wang C., Chen P.-X., Xiao Q., Chen J., Chen F.-Q., Yang Q.-M., Weng H.-F., Fang B.-S., Zhang Y.-H., Xiao A.-F. (2022). Artificial naringinase system for cooperative enzymatic synthesis of naringenin. Biochem. Eng. J..

[45] Koseki T., Mese Y., Nishibori N., Masaki K., Fujii T., Handa T., Yamane Y., Shiono Y., Murayama T., Iefuji H. (2008). Characterization of an α-L-rhamnosidase from *Aspergillus kawachii* and its gene. Biochem. Relev. Enzym. Proteins.

[46] Thammawat K., Pongtanya P. (2008). Isolation, Preliminary Enzyme Characterization and Optimization of Culture Parameters for Production of Naringinase Isolated from *Aspergillus niger* BCC 25166. Nat. Sci..

[47] Gallego M.V., Pinaga F., Ramon D., Valles S. (2001). Purification and characterization of an alpha-L-rhamnosidase from *Aspergillus terreus* of interest in winemaking. J. Food Sci..

[48] Decker C.H., Visser J., Schreier P. (2000). B-Glucosidases from Five Black *Aspergillus* Species: Study of Their Physico-Chemical and Biocatalytic Properties Keywords: *J*. Agric. Food Chem..

[49] Muñoz M., Holtheuer J., Wilson L., Urrutia P. (2022). Grapefruit Debittering by Simultaneous Naringin Hydrolysis and Limonin Adsorption Using Naringinase Immobilized in Agarose Supports. Molecules.

[50] Zhu Y., Jia H., Xi M., Li J., Yang L., Li X. (2017). Characterization of a naringinase from *Aspergillus oryzae* 11250 and its application in the debitterization of orange juice. Process Biochem..

[51] Roitner M., Schalkhammer T., Pittner F. (1984). Characterisation of naringinase from *Aspergillus niger*. Monatshefte Für Chem. Chem. Mon..

[52] Manzanares P., Hetty C., Visser J. (2001). Purification and Characterization of Two Different-alpha-L-Rhamnosidases, RhaA and RhaB, from *Aspergillus aculeatus*. Appl. Environ. Microbiol..

[53] Abbate E., Palmeri R., Todaro A., Blanco R.M. (2012). Production of a α-L-Rhamnosidase from *Aspergillus terreus* Using Citrus Solid Waste as Inducer for Application in Juice Industry. Chem. Eng. Trans..

[54] Chang H.-Y., Lee Y.-B., Bae H.-A., Huh J.-Y., Nam S.-H., Sohn H.-S., Lee H.J., Lee S.-B. (2011). Purification and characterisation of *Aspergillus sojae* naringinase: The production of prunin exhibiting markedly enhanced solubility with in vitro inhibition of HMG-CoA reductase. Food Chem..

[55] Zdarta J., Jędrzak A., Klapiszewski Ł., Jesionowski T. (2017). Immobilization of Cellulase on a Functional Inorganic–Organic Hybrid Support: Stability and Kinetic Study. Catalysts.

[56] Spagna G., Barbagallo R.N., Casarini D., Pifferi P.G. (2001). A novel chitosan derivative to immobilize alpha-L-rhamnopyranosidase from Aspergillus niger for application in beverage technologies. Enzym. Microb. Technol..

[57] (2014). Keerti; Gupta, A.; Kumar, V.; Dubey, A.; Verma, A.K. Kinetic Characterization and Effect of Immobilized Thermostable beta -Glucosidase in Alginate Gel Beads on Sugarcane Juice. ISRN Biochem..

[58] Spagna G., Romagnoli D., Angela M., Bianchi G., Pifferi P.G. (1998). A simple method for purifying glycosidases: Alpha-L-arabinofuranosidase and beta-D-glucopyranosidase from Aspergillus niger to increase the aroma of wine. Part, I. Enzym. Microb. Technol..

